# First detection and characterization of a non-haemadsorbing African swine fever virus (genotype II) isolated from a wild boar in Russia

**DOI:** 10.3389/fmicb.2026.1907676

**Published:** 2026-09-18

**Authors:** R. S. Chernyshev, A. S. Sadchikova, D. S. Moiseenko, A. R. Shotin, I. A. Lavrentiev, I. S. Kolbin, N. G. Zinyakov, A. S. Igolkin

**Affiliations:** Federal Centre for Animal Health (ARRIAH), Vladimir, Russia

**Keywords:** ASFV, genotype II, moderate virulence, non-haemadsorbing (non-HAD), Russia

## Abstract

In 2023, a non-haemadsorbing African swine fever virus (ASFV) of genotype II (isolate ASFV/Kirov/WB2023/1448) was recovered from a wild boar in the Kirov Region of Russia—the first such case during the current epidemic (2007–present). Whole-genome analysis of the non-haemadsorbing ASFV revealed a large deletion (~1,300 base pairs) in the *EP153R* and *EP402R* genes, and a smaller deletion (~50 base pairs) in the *MGF 110-1 L* gene. Phylogenetic analysis confirmed that ASFV/Kirov/WB2023/1448 belongs to the genetic lineage «II/EU1/RU7», which is typical for the European part of Russia. Following intramuscular inoculation of eight domestic pigs with isolate ASFV/Kirov/WB2023/1448 at a dose of 10^3^ TCID_50_ moderate virulence was observed, with the development of acute and subacute forms of ASF. Furthermore, the non-haemadsorbing virus was shown to be contagious, as two pigs maintained in direct contact with the inoculated animals developed ASF. The mortality rate among pigs infected via the intramuscular and contact routes was 60%. Two out of ten infected pigs recovered from ASF; persistence of the non-haemadsorbing virus was not detected, as complete elimination of the virus from the host organism was observed within 28 days post-infection. Upon challenge of the recovered pigs with the highly virulent haemadsorbing isolate ASFV/Kirov/WB2023/1447—which had been recovered from the same wild boar population as ASFV/Kirov/WB2023/1448—the animals exhibited no clinical signs of disease, viraemia, virus shedding, or virus dissemination in organs and tissues. To investigate the possible circulation of the non-haemadsorbing virus in wildlife, 122 field samples collected from hunted and found-dead wild boars within a single population in Kirov Region and adjacent regions of the Russian Federation during the period 2021–2023 were examined. Two hunted animals sampled in 2023 in the Republic of Mari El (bordering Kirov Region) were found to be seropositive with no detectable ASFV. The present study may indicate the emergence of natural mechanisms of virulence attenuation of ASFV genotype II in the European wild boar population over a relatively short period of pathogen circulation (15–16 years).

## Introduction

The highly virulent African swine fever virus (ASFV) of genotype II has continued to circulate across many countries in Europe and Asia since its introduction into Georgia in 2007 (Beltrán-Alcrudo et al., 2008; European Food Safety Authority (EFSA) et al., 2025; Mighell and Ward, 2021). The European wild boar (*Sus scrofa scrofa*) is a key natural reservoir of ASFV, maintaining its spread in wildlife and facilitating periodic spillover into domestic pig populations (*Sus scrofa domesticus*) (Zakharova et al., 2023). Since 2014, ASF outbreaks have been predominantly reported in wild boar populations in many European countries, including Poland, Lithuania, Latvia, Estonia, Bulgaria, Hungary, Germany, Italy, and others (Sauter-Louis et al., 2021; Pavone et al., 2023). Global experience in ASF control demonstrates that virus circulation in areas with high wild boar population density (e.g., the Baltic States) leads to a reduction in ASFV virulence. Consequently, chronic and asymptomatic disease courses are increasingly reported, which significantly complicates diagnostic and eradication efforts (Gallardo et al., 2019). Prolonged circulation of the virus among susceptible animal populations can drive mutational processes, including deletions of individual genes or large genomic regions. Consequently, whole-genome analysis and characterization of the biological properties of newly identified isolates are critical components of epidemiological surveillance. These approaches also offer a promising avenue for identifying naturally attenuated ASFV strains—potential intermediate candidates for the development of live vaccines (Barasona et al., 2021).

ASFV contains double-stranded DNA and is the only known DNA arbovirus, belonging to the genus *Asfivirus* within the family *Asfarviridae* (Gaudreault et al., 2020). The genome of the Georgia 2007/1 strain (genotype II) spans 190,584 kilobase pairs (kbp), comprises 195 open reading frames (ORFs), and encodes over 150 proteins (Chapman et al., 2011). ASFV can enter target cells—primarily porcine mononuclear cells and endothelial cells—via multiple pathways, including clathrin-dependent endocytosis, macropinocytosis, and efferocytosis (the uptake of apoptotic bodies by macrophages) (Dixon, 2025). This diversity of entry mechanisms, combined with the virus’s large genome and high number of open reading frames (ORFs), contributes to the presence of multiple virulence factors. The principal virulence determinants are encoded within five multigene families (MGFs): MGF 100, MGF 110, MGF 300, MGF 360, and MGF 505. These MGFs are located in the variable terminal regions of the genome (Zhu et al., 2021), where most insertions and deletions (indels) have been documented (Senthilkumar et al., 2022). Furthermore, microhomology among different ORFs within the same family can facilitate non-allelic homologous recombination, leading to large-scale genomic alterations such as deletions or duplications (Fiori et al., 2022).

Additionally, the CD2v glycoprotein—encoded by the *EP402R* gene—represents a crucial virulence factor in ASFV, as it is responsible for haemadsorption (Rodríguez et al., 1993). This phenomenon is of significant importance for virological diagnosis, since most genotype II isolates retain the ability to attach erythrocytes to the surface of infected cells (de León et al., 2013). The World Organization for Animal Health (WOAH) recognizes virus isolation in susceptible cell cultures followed by haemadsorption test (HAD) as a reference method for ASFV diagnosis—the so-called “gold standard” (WOAH—World Organization for Animal Health, 2025). Notably, haemadsorption observed in primary cultures of porcine mononuclear cells serves as a phenotypic marker of genetic changes in the *EP402R* gene. Consequently, the presence or absence of haemadsorption is a key characteristic of the cultural (reproductive) properties of ASFV isolates and is typically the first trait assessed during monitoring of ASFV variants. Moreover, CD2v idiotypes form the basis of seroimmunotyping. Currently, nine classified seroimmunotypes have been identified through immunological (IT) and haemadsorption inhibition tests (HAdI) using reference sera (Sereda et al., 2023; Malogolovkin and Sereda, 2022). To date, all known genotype II strains and isolates divergent from Georgia 2007/1 belong to seroimmunotype VIII (Malogolovkin et al., 2015). Numerous studies have demonstrated a positive correlation between attenuation of ASFV and loss of haemadsorbing activity (Yang et al., 2024; Teklue et al., 2020; Barasona et al., 2026). For example, pigs immunized with the genetically modified BA71ΔCD2 variant (genotype I), carrying a deletion in the *EP402R* gene (at a dose of 10^6^ TCID_50_), showed no clinical signs of ASF and were protected upon challenge infection with both the homologous E75 (genotype I) and heterologous strains Georgia 2007/1, RSA/11/2017 (genotype XIX), and Ken06. Bus (genotype IX) (Monteagudo et al., 2017; Lopez et al., 2020).

In field conditions, non-haemadsorbing variants of the ASFV were isolated both from biotopes of soft ticks of the genus *Ornithodoros* and from domestic pigs and wild boars.

Notably, the NH/P68 and OURT88/3 strains (genotype I), isolated in the Iberian Peninsula (Portugal) from domestic pigs and *Ornithodoros erraticus* ticks respectively, exhibited low virulence. These strains caused chronic or asymptomatic ASF courses and induced protection against infection with highly virulent genotype I ASFV (Boinas et al., 2004; Leitão et al., 2001; Sánchez-Cordón et al., 2017). However, their natural origin has been questioned. A hypothesis suggests that these strains may have originated from an attenuated cell-culture ASFV of genotype I, which was used in live vaccines in Portugal during the second half of the 20th century (Portugal et al., 2015).

Since the emergence of genotype II ASFV in Eurasia (2007–present), several cases of non-haemadsorbing variants in wild boars have been documented. A key example is the Lv17/WB/Rie1 strain, isolated and characterized in Latvia in 2017 (Gallardo et al., 2019; Gallardo et al., 2024; González-García et al., 2025). This strain serves as a prototype in the development of an experimental ASF vaccine within the EU project VACDIVA. Experimental inoculation of pigs with the Lv17/WB/Rie1 strain resulted in asymptomatic and chronic disease courses, characterized by persistent viraemia and widespread viral dissemination across most organs and tissues over 45–94 days post-infection. In a challenge infection study, two pigs were exposed to a haemadsorbing genotype II ASFV on day 58 post-inoculation. One animal developed clinical signs and viraemia, while the other remained clinically healthy but exhibited persistent viraemia (Gallardo et al., 2019). Importantly, oral immunization of wild boars with the Lv17/WB/Rie1 strain provided 92% protection against challenge infection with the Arm 07 strain (genotype II) (Barasona et al., 2019). The safety of a vaccine candidate based on this strain is currently being evaluated in wild boar populations as part of ongoing EU efforts (Barasona et al., 2021; González-García et al., 2025).

Another significant development is the identification of 11 non-haemadsorbing ASFV genotype II isolates from domestic pigs in Heilongjiang and Hubei provinces of China (2020) (Sun et al., 2021). All isolates originated from the initial highly virulent variant responsible for the first ASFV outbreaks in China (strain HLJ/18). Experimental infection studies revealed dose-dependent outcomes: at a high dose (10^6^ TCID_50_), mortality (50–75%), acute, and subacute disease courses were observed; at a low dose (10^3^ TCID_50_), subacute and chronic courses with prolonged viral persistence and no mortality were recorded. Thus, the described Chinese non-haemadsorbing ASFV corresponds to moderately virulent and low-virulent variants.

This study presents the characterization of the biological properties of a non-haemadsorbing genotype II ASFV (isolate ASFV/Kirov/WB2023/1448), first recovered from a wild boar through laboratory investigations in Russia. To investigate the circulation of the non-haemadsorbing variant within the studied population, the results of the analysis of biological material obtained from wild boars—both found dead and hunted—in Kirov and adjacent regions of the Russian Federation (2021–2023) are also presented.

## Materials and methods

### Samples

The original *samples* were submitted to the Reference Laboratory for ASF of the Federal Centre for Animal Health (FGBI «ARRIAH», Vladimir, Russia) as part of reference studies and ASF epidemiological surveillance. During the period from 2012 to 2023, routine virus isolation from submitted samples revealed, for the first time, a non-haemadsorbing isolate in sample No. 1448, collected from a hunted wild boar in July 2023 in Kirov Region. Concurrently, within the same wild boar population in Kirov Region in July 2023, a haemadsorbing ASFV isolate was recovered from sample No. 1447; the biological properties of this isolate are also reported herein as a reference.

To investigate the circulation of the non-haemadsorbing virus in the population, this study also presents the results of testing 122 samples from found-dead and hunted wild boars collected in Kirov and adjacent regions of the Russian Federation during 2021–2023, using real-time polymerase chain reaction (PCR), immunoperoxidase test (IPT), and virus isolation.

Samples were prepared prior to analysis. Serum samples were clarified by centrifugation at 850 × g for 10 min. Ten percent (10.0%) suspensions of biological material (blood, spleen, liver, lymph nodes) were prepared in sterile physiological saline using an automated homogenizer, followed by centrifugation at 850 × g for 30 min.

The clarified suspensions and sera were passed through a 0.45 μm pore-size filter and used for IPT, real-time PCR, and virus isolation.

### Cell culture

For ASFV isolation and cultivation, a primary culture of porcine alveolar macrophages (PAM) was established using clinically healthy piglets aged 4–6 weeks (body weight: 8–15 kg) that were free from ASFV and other infectious diseases as donors. Euthanasia and lung collection were performed in accordance with Directive 2010/63/EU of the European Parliament and of the Council of 22 September 2010 on the protection of animals used for scientific purposes. Isolated lungs were placed in a sterile container and transported to the laboratory, where all subsequent manipulations were carried out under a laminar airflow hood. The procedure began with tracheal treatment: 10.0 mL of a 20.0% ethanol solution was introduced into the tracheal lumen, incubated for 5 min, and then removed. Subsequently, bronchoalveolar lavage was performed by filling the lungs with 600.0 mL of saline (0.9% sodium chloride solution) supplemented with 1.6 mg/mL gentamicin sulfate, 100 μg/mL streptomycin sulfate, and 1,000 IU benzylpenicillin sodium salt. After a 10-min incubation, the fluid was aspirated and centrifuged at 4 °C and 1,500 g for 20 min to pellet the cells. The supernatant was discarded, and the cell pellet was resuspended in 100.0 mL of saline containing 50,000 IU amphotericin B, followed by a second centrifugation at the same conditions (4 °C, 1500 g, 20 min). After removal of the supernatant, the cells were resuspended in RPMI-1640 growth medium (Himedia, United States) supplemented with 15.0% fetal bovine serum (FBS, SERANA, Brazil), 0.08% gentamicin sulfate, L-glutamine, and sodium bicarbonate. The cell suspension was then passed through a 40 μm strainer (SPL Life Sciences, South Korea), and the concentration was determined using an automated Countess™ cell counter (Invitrogen, United States) with a selective protocol for cells sized 20–40 μm. The suspension was adjusted to a seeding concentration of 350 × 10^3^ cells/ml in RPMI-1640 medium and seeded into T-25 culture flasks and 96-well plates (SPL Life Sciences, South Korea). The cells were incubated at 37 °C in 5.0% CO_2_, and after 48 h, the conditioning medium was replaced with maintenance RPMI-1640 medium supplemented with 8.0% fetal bovine serum and 0.001% gentamicin sulfate. When a satisfactory subconfluent monolayer was achieved, the PAM culture was used for ASFV infection.

### Virus isolation

The filtrate of the suspensions or sera (1.0 mL per sample) was inoculated onto a sub-confluent monolayer of PAM cell culture («blind» passage). After 30 min of virus adsorption at 37 °C, 9.0 mL of maintenance medium was added to the culture flasks, and infected cells was incubated at 37 °C until complete macrophage disaggregation occurred. The resulting virus-containing suspension was subjected to freeze–thaw cycles and centrifugation at 3000 g for 5 min to obtain a supernatant for subsequent passages. Virus replication dynamics were studied over 10 consecutive passages. For virus titration, ten-fold dilutions in RPMI-1640 medium were prepared and inoculated onto subconfluent PAM monolayers (12 replicates per dilution), followed by 6 days of incubation at 37 °C and 5.0% CO₂. Virus titer was calculated using the Kerber method, and results were detected by haemadsorption test (HAD) and real-time PCR.

### Real-time PCR

DNA extraction was performed using the «DNeasy Blood & Tissue Kit» (QIAGEN, Germany) according to the manufacturer’s protocol, and real-time PCR was conducted following the method described by Fernández-Pinero et al. (2013), recommended by the WOAH (WOAH—World Organization for Animal Health, 2025; Fernández-Pinero et al., 2013). The «Platinum II Taq Hot-Start DNA Polymerase» kit (ThermoFisher Scientific, United States) was used for PCR mix preparation, and semi-quantitative analysis of real-time PCR results was performed. A sample was considered positive if the cycle threshold (Ct) value did not exceed 40.

### Enzyme-linked immunosorbent assay

The serological status of animals was determined using the blocking ELISA kit «INGEZIM PPA COMPAC» (Ingenasa, Spain) for detection of ASFV-specific antibodies in pig serum, while the *indirect immunoperoxidase test* (IPT) was performed according to WOAH recommendations (WOAH—World Organization for Animal Health, 2025).

### Direct immunofluorescence assay

Additional detection of ASFV replication was performed by DIF. PAM culture was infected with ASFV at a multiplicity of infection (MOI) of 0.1 TCID_50_ or 0.1 HAD_50_ and incubated for 24 h at 37 °C and 5.0% CO_2_. The subconfluent cell monolayer was washed three times with 10.0% phosphate-buffered saline (PBS), and cells were fixed with a 1:1 mixture of 95.0% ethanol and acetone for 2 min, then air-dried at room temperature. Infected cells were stained with FITC-conjugated immunoglobulin against ASFV vp72 protein (Ingenasa, Spain; 1:50 dilution in nuclease-free water) for 30 min, washed three times with 10% PBS, and air-dried. Reaction visualization was performed using fluorescence microscopy.

### Whole-genome sequencing, assembly and alignment

For whole-genome sequencing, genomic DNA preparation was performed according to a previously described protocol (Chernyshev et al., 2024), and library preparation was carried out using the «MGIEasy Universal DNA Library Prep Set» (MGI Tech, China). High-throughput sequencing was performed on the DNBSEQ-G50 platform (MGI Tech). Consensus assembly was achieved by mapping reads to the reference genome of the Georgia 2007/1 strain (NC_044959.2) using Unipro UGENE, while variant detection was performed using SnapGene v. 5.2.1. The sequences of the two isolates were annotated and deposited in the GenBank database. Accession numbers: PZ412709 (ASFV/Kirov/WB2023/1448) and PZ412710 (ASFV/Kirov/WB2023/1447). Genotyping was performed according to Bastos et al. (2003).

*Phylogenetic analysis* involved multiple sequence alignment of isolates using MAFFT (Galaxy Version 7.526 + galaxy2), with comparative analysis including strains and isolates collected in Eurasian countries between 2007 and 2023. Phylogeny was reconstructed using Mega X with the Tamura model and gamma distribution (T92 + G), selected based on Bayesian information criterion (BIC) and corrected Akaike information criterion (AICc) values using the maximum likelihood (ML) method. Visualization of the dendrogram was performed using the online platform iTOL (Letunic and Bork, 2024), and determination of topotypes and genetic lineages was performed according to a previously proposed classification (Chernyshev et al., 2026).

### Biological experiment

For the *biological experiment* aimed at assessing the biological properties of the non-haemadsorbing isolate ASFV/Kirov/WB2023/1448, a challenge study was conducted using domestic pigs imported from farms free of major swine infectious diseases. The experiment complied with institutional and national standards for the use of laboratory animals, in accordance with Directive 2010/63/EU of the European Parliament and of the Council of 22 September 2010 on the protection of animals used for scientific purposes. The study involved 12 Large White pigs aged 2–2.5 months with a body weight of 15–20 kg. The animals were housed in isolated biosafety level 3 (BSL-3) facilities at ARRIAH (Vladimir, Russia), specifically designed for work with the ASFV pathogen.

The detailed experimental design is presented in Figure 1.

**Figure 1:**
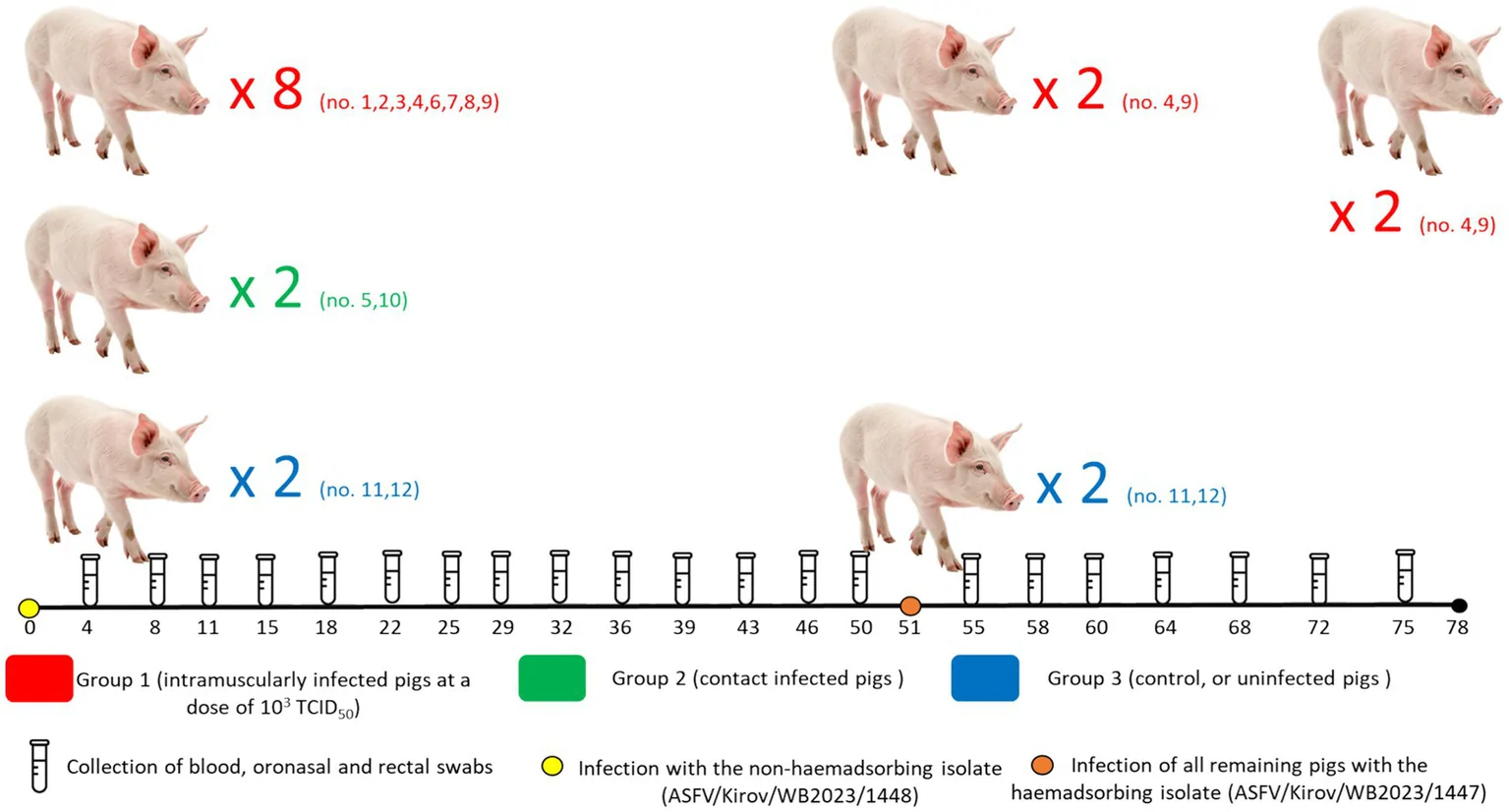
The schematic of the biological experiment to assess the biological properties of the ASFV isolate ASFV/Kirov/WB2023/1448 in domestic pigs is presented; the days marked on the scale correspond to days post-infection (dpi).

Following quarantine, the pigs were randomly assigned to three groups: Group 1 (intramuscularly inoculated pigs no. 1, 2, 3, 4, 6, 7, 8, 9), Group 2 (contact-infected pigs no. 5, 10), and a control group (pigs no. 11, 12). Pigs in Group 1 were inoculated intramuscularly with the isolate ASFV/Kirov/WB2023/1448 at a dose of 10^3^ TCID_50_ (1.0 mL). Intact animals in Group 2 were housed together with the intramuscularly inoculated pigs to assess the possibility of contact transmission. Pigs in the control group were inoculated intramuscularly with ASFV-free PAM cell culture suspension (1.0 mL) and housed in a separate room. Following the resolution of viraemia, all surviving pigs (recovered and control) were challenged with the haemadsorbing isolate ASFV/Kirov/WB2023/1447 at a dose of 10^3^ HAD_50_ (1.0 mL) administered intramuscularly.

Animals were monitored daily; clinical signs were assessed visually, and rectal body temperature was recorded for each pig.

Oronasal and rectal swabs (using disposable swab probes) were collected from each animal twice weekly (at 3–4-day intervals) from the onset of the experiment until death or euthanasia. Following collection, pooled oronasal and rectal swabs (in physiological saline) were prepared for each pig and analyzed by real-time PCR. Blood samples (5.0 mL) were collected into tubes containing a clotting activator (sodium citrate) from each pig twice weekly until death or euthanasia. Blood samples were separated into clot and serum fractions. Clots were analyzed by real-time PCR, and sera were tested by ELISA. On the day of death or euthanasia, necropsy was performed, and 10 tissue samples were collected from each carcass (one sample each of spleen, kidneys, liver, lung, bone marrow, muscle tissue, synovial membrane, and submandibular, inguinal, and mesenteric lymph nodes) and analyzed by real-time PCR. Euthanasia of the pigs was performed by intravenous administration of pentobarbital. The drug was given at a concentration of 400 mg/mL, with a volume of 0.1 mL administered per kilogram of the animal’s live body weight.

### Statistical analysis

Comparison of two independent samples for a quantitative variable (virus titre or Ct value between two consecutive passages) was performed using the Mann–Whitney U test. Absolute values were automatically converted to ranks. Differences between samples were considered statistically significant when the U-statistic fell within the «green» (*p* ≤ 0.01) or «grey» (*p* ≤ 0.05) zone; differences were considered statistically non-significant when the U-statistic fell within the «red» (*p* > 0.05) zone. Differences were considered statistically non-significant at *p* > 0.05.

Graphs were generated using GraphPad Prism v.8.0.

## Results

### Reproductive (cultural) properties

For comparative analysis, data for two isolates are presented: the non-haemadsorbing ASFV/Kirov/WB2023/1448 (the main subject of the study) and the haemadsorbing ASFV/Kirov/WB2023/1447 (used as a reference isolate; it was recovered from the same wild boar population at the same time as the non-haemadsorbing isolate). During 10 consecutive passages in PAM cell culture, a stable viral accumulation (plateau effect) was observed, which was consistent for both isolates throughout the entire cultivation period. As shown in Figures 2A,B, differences in viral titer values and Ct values between the haemadsorbing and non-haemadsorbing ASFV isolates were statistically non-significant (*p* > 0.05) across passages 1–10. Serial passage of ASFV/Kirov/WB2023/1448 in PAM did not result in the emergence of haemadsorption, indicating the homogeneity of the viral population. In contrast, the ASFV/Kirov/WB2023/1447 isolate exhibited the haemadsorption typical of ASFV throughout passages 1–10 (Figures 2C,D). Despite the absence of haemadsorption, the ASFV/Kirov/WB2023/1448 isolate induced a cytopathic effect (CPE) in PAM. The CPE manifested as vacuolisation, cellular destruction, disaggregation, and lysis of macrophages 48–96 h post-infection (hpi) (Figures 2E,F). The presence of specific antigen in PAM infected with the non-haemadsorbing virus was also confirmed by DIF 24 hpi, prior to the onset of CPE (Figures 2G,H).

**Figure 2:**
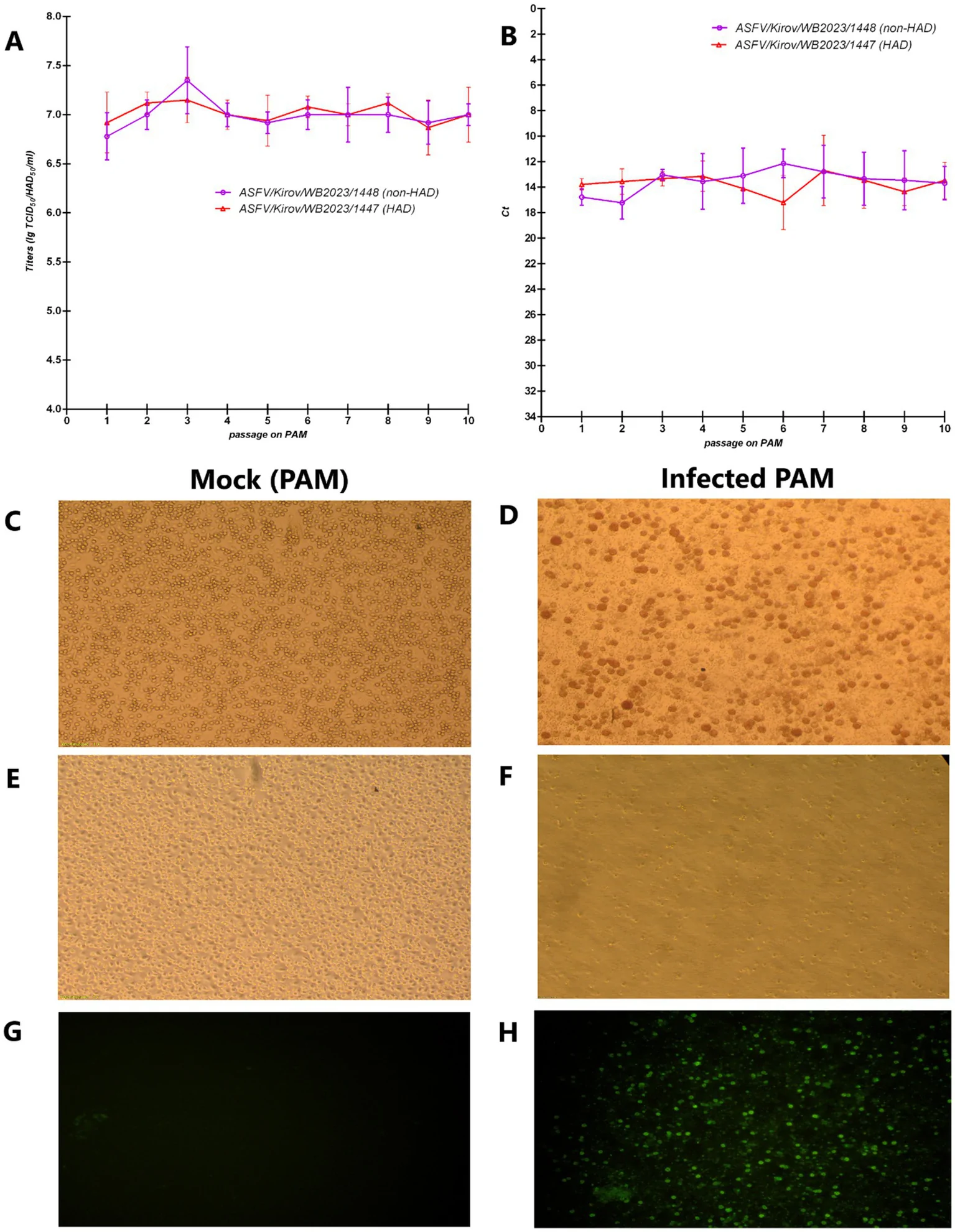
Cultural properties of ASFV isolates recovered in the Kirov Region, Russia (2023): **(A)** Dynamics of viral accumulation for the haemadsorbing (HAD) and non-haemadsorbing (non-HAD) isolates in PAM cell culture over passages 1–10, expressed as viral titres TCID_50_/HAD_50_/mL; **(B)** Semi-quantitative assessment of viral accumulation dynamics for both isolates in PAM across passages 1–10, based on real-time PCR results (*Ct*—Cycle threshold of amplification); **(C,E)** Mock (intact PAM at 96 h post-seeding); **(D)** Haemadsorption phenomenon of the ASFV/Kirov/WB2023/1447 isolate; **(F)** CPE in PAM at 72 h post-infection with the ASFV/Kirov/WB2023/1448 isolate; **(G)** Mock (intact PAM cell culture after staining with FITC-conjugated immunoglobulin against ASFV vp72 protein at 72 h post-seeding); **(H)** PAM at 24 h post-infection with the ASFV/Kirov/WB2023/1448 isolate, followed by staining with FITC-conjugated immunoglobulin against ASFV vp72 protein.

### Whole-genome analysis

During reference-based genome assembly, a non-covered region (a deletion of approximately 1,300 bp) was identified in the *EP153R* and *EP402R* genes of the ASFV/Kirov/WB2023/1448 isolate, located at positions 74.0–75.3 kbp relative to the Georgia 2007/1 strain (Figure 3A). Since the *EP402R* gene encodes the CD2v glycoprotein, which is responsible for haemadsorption, this finding is consistent with the cultual properties of the non-haemadsorbing virus. Additionally, another non-covered region (a 50 bp deletion) was detected in the *MGF 110-1 L* gene sequence of the same isolate, located at positions 7.0–7.05 kbp relative to the Georgia 2007/1 strain (Figure 3B). The remainder of the ASFV/Kirov/WB2023/1448 genome was uniformly and completely covered, indicating the absence of other deletions. In contrast, the genome assembly of the haemadsorbing isolate ASFV/Kirov/WB2023/1447 achieved 100% coverage (Figure 3C).

**Figure 3:**
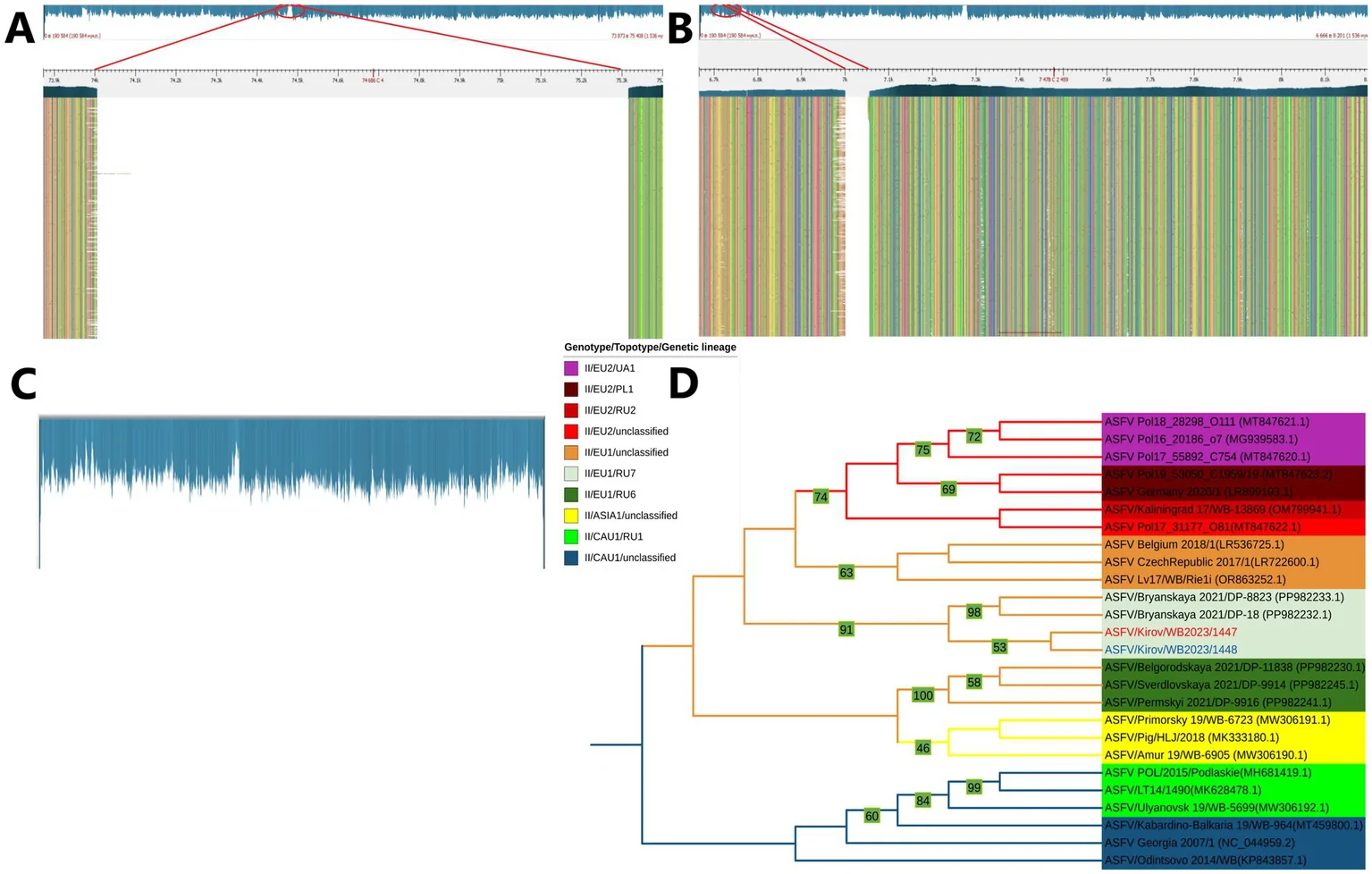
Molecular properties of ASFV isolates recovered in the Kirov Region, Russia (2023): **(A)** An uncovered region (lacking aligned reads mapped to the reference) identified during the assembly of the non-haemadsorbing isolate ASFV/Kirov/WB2023/1448. This region is located at positions 74.0–75.3 kbp relative to the Georgia 2007/1 strain, within the *EP153R* и *EP402R* genes; **(B)** An uncovered region (lacking aligned reads mapped to the reference) detected during the assembly of the non-haemadsorbing isolate ASFV/Kirov/WB2023/1448, located at positions 7.0–7.05 kbp relative to the Georgia 2007/1 strain, in the *MGF 110-1 L* gene; **(C)** 100% genome coverage achieved during the assembly of the haemadsorbing isolate ASFV/Kirov/WB2023/1447; **(D)** A cladogram depicting the phylogenetic relationships among ASFV isolates of genotype II obtained in Eurasia. The branch color corresponds to the topotype, while the leaf color corresponds to the genetic lineage. The haemadsorbing isolate ASFV/Kirov/WB2023/1447 is highlighted in red, and the non-haemadsorbing isolate ASFV/Kirov/WB2023/1448 is highlighted in blue.

Analysis of the *B646L* locus in both studied isolates revealed genotype II.

Following multiple sequence alignment with known ASFV strains of genotype II isolated during the current epidemic in Eurasia, phylogenetic analysis demonstrated that both isolates belong to the genetic lineage «II/EU1/RU7», which is typical of the European part of Russia (Figure 3D). The ASFV/Kirov/WB2023/1447 and ASFV/Kirov/WB2023/1448 isolates are classified into genetic variants based on specific single-nucleotide polymorphisms (SNPs), including I267L-II (T → A at position 170,862 relative to the Georgia 2007/1 strain), MGF 360-15R-II (G → A at positions 50,655 and 50,667), MGF 505-10R-II (G → A at position 46,557), NP1450L-II (G → C at position 131,463), and Q706L-II (C → G at position 158,805). These SNPs confirm their affiliation with the «II/EU1/RU7» lineage (Letunic and Bork, 2024). Both isolates from the Kirov Region form a subclade (sub-lineage) within this genetic lineage, indicating a common origin.

### Immunobiological properties

Prior to challenge with the haemadsorbing isolate ASFV/Kirov/WB2023/1447, the pigs in the control group (no. 11 and 12) were clinically healthy.

The incubation period following infection with the ASFV/Kirov/WB2023/1448 isolate in pigs from groups 1 and 2 ranged from 7 to 11 days post-infection (dpi). The duration of the disease after the onset of the first clinical signs ranged from 4 to 29 dpi in both groups, which is typical for acute and subacute courses of ASF. Remittent or recurrent fever was recorded in all infected animals (Figure 4A). Throughout the entire observation period, animals exhibited clinical signs typical of ASF, including reduced appetite progressing to complete anorexia and dehydration, neurological involvement ranging from asthenia to paralysis, respiratory signs such as dyspnea, cough, and catarrhal nasal discharge, gastrointestinal signs such as diarrhea, cyanotic skin areas up to 15% of the total skin surface, serous-purulent conjunctivitis, and arthritis.

**Figure 4:**
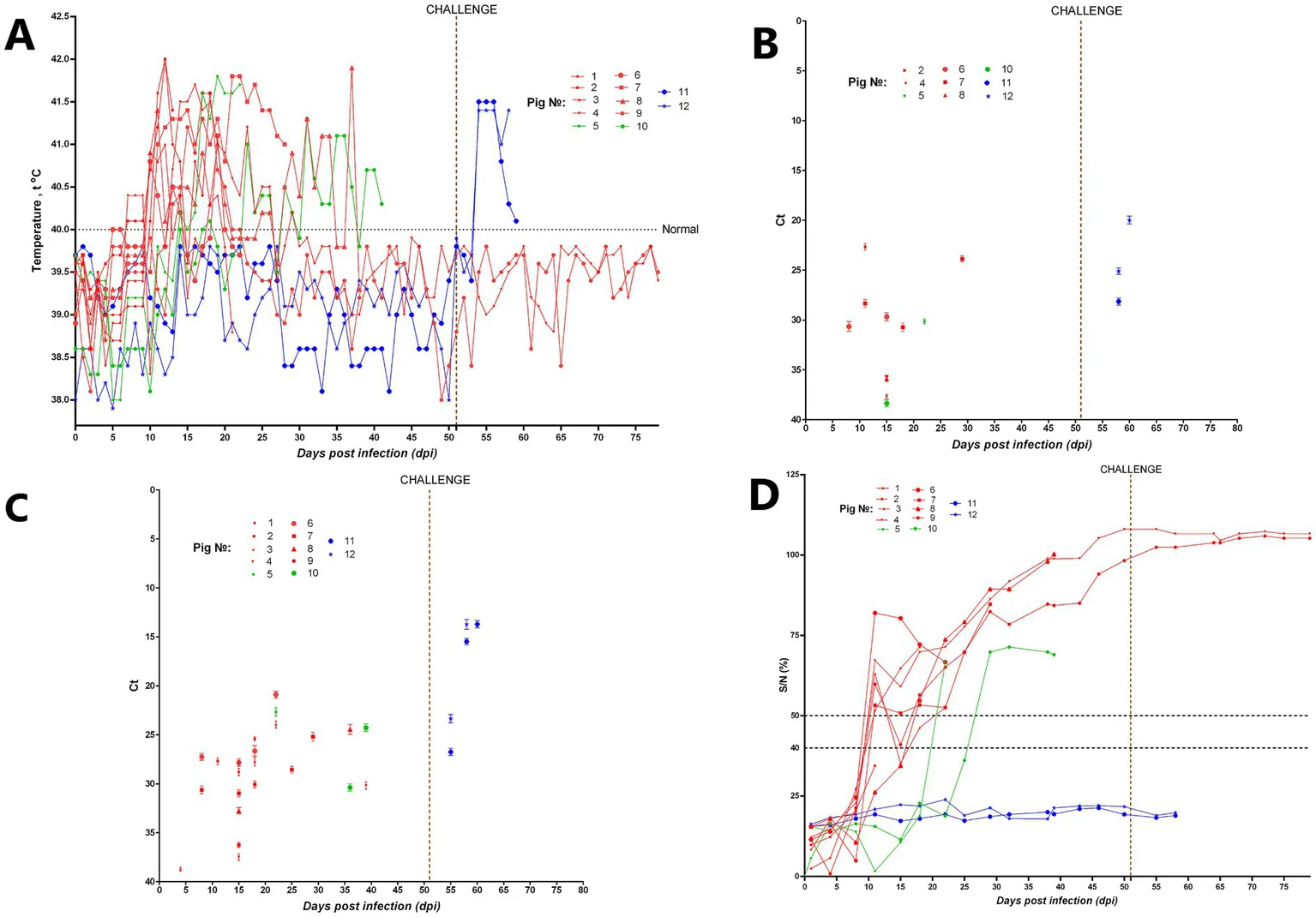
Characteristics of pathogenesis in domestic pigs infected with the non-haemadsorbing isolate ASFV/Kirov/WB2023/1448. Signatures for intramuscularly infected pigs are marked in red, for contact-infected pigs in green, and for control pigs in blue. **(A)** Body temperature in pigs over the 78-day experimental period. **(B)** The dynamics of viral shedding in pigs, detected by real-time PCR of oronasal and rectal swabs. **(C)** The pattern of viraemia in pigs, as detected by real-time PCR. **(D)** The period of seroconversion and the dynamics of antibody production in pigs, determined in blood sera by enzyme-linked immunosorbent assay (ELISA). The S/N ratio (sample optical density divided by inhibition control optical density, expressed as a percentage). The lower dashed line indicates the threshold for a ‘equivocal’ result, and the upper dashed line indicates the threshold for a ‘positive’ result.

All infected pigs were additionally tested for viral shedding by real-time PCR of oronasal and rectal swabs. As shown in Figure 4B, shedding of ASFV from infected animals occurred from 7 to 28 dpi. Viaraemia dynamics in infected pigs was also assessed by real-time PCR. As illustrated in Figure 4C, the infectious process was characterized by low viral load in blood, with Ct values ranging from 20.89 to 39.70. The longest duration of viraemia was observed in pig no. 7 (21 days). No persistent viraemia was detected. Seroconversion and antibody response dynamics were monitored throughout the experiment (Figure 4D). According to the results, seroconversion was observed in intramuscularly and contact-infected animals with the non-haemadsorbing isolate ASFV/Kirov/WB2023/1448, except for pig no. 2.

Mortality in pigs infected with the non-haemadsorbing ASFV was 60%: pigs no. 1, 2, 5, 6, 7, 10 died; pigs no. 3 and 8 were euthanized; pigs no. 4 and 9 recovered and survived. The pigs subjected to euthanasia did not affect the mortality rate, as they were humanely euthanized due to clinical signs of secondary infections. It is noteworthy that contact-infected pigs no. 5 and 10 died on days 21–40 and exhibited typical pathological changes of ASF, including splenomegaly, hemorrhagic syndrome, and generalized lymphadenitis. The same lesions were observed at necropsy of intramuscularly infected pigs no. 1 and 2. In the remaining animals (no. 3, 6, 7, 8), signs of secondary infections (pasteurellosis, streptococcosis, and Glasser’s disease) were noted, including fibrinopurulent arthritis, fibrinous pleuropneumonia, pericarditis, and peritonitis.

On 51 dpi, the recovered pigs (no. 4, 9) and control pigs (no. 11, 12) were challenged with the haemadsorbing isolate ASFV/Kirov/WB2023/1447. Over the subsequent 28-day observation period following the challenge infection, pigs no. 4 and 9 showed no signs of fever, viraemia, or viral shedding; they remained clinically healthy. These animals were subsequently subjected to diagnostic slaughter and organ examination for the presence of ASFV. In contrast, pigs from the control group (no. 11, 12) exhibited a typical acute course of ASF lasting 4–5 days, characterized by high viral load in blood and lethal outcome. Notably, no seroconversion was observed in these animals (Figures 4A–D).

Post-mortem examination of pigs no. 4 and 9 revealed no pathological changes typical of ASF. In pigs no. 11 and 12, however, the following lesions were recorded: splenomegaly, generalized hemorrhagic lymphadenitis, pulmonary oedema, hemorrhages in the renal cortex, under the epicardium, and in the liver (Figures 5A–D). Real-time PCR of samples collected from all 12 pigs confirmed the absence of ASFV in organs and tissues of the recovered pigs no. 4 and 9. In contrast, all other animals exhibited total viral dissemination (Figure 5E).

**Figure 5:**
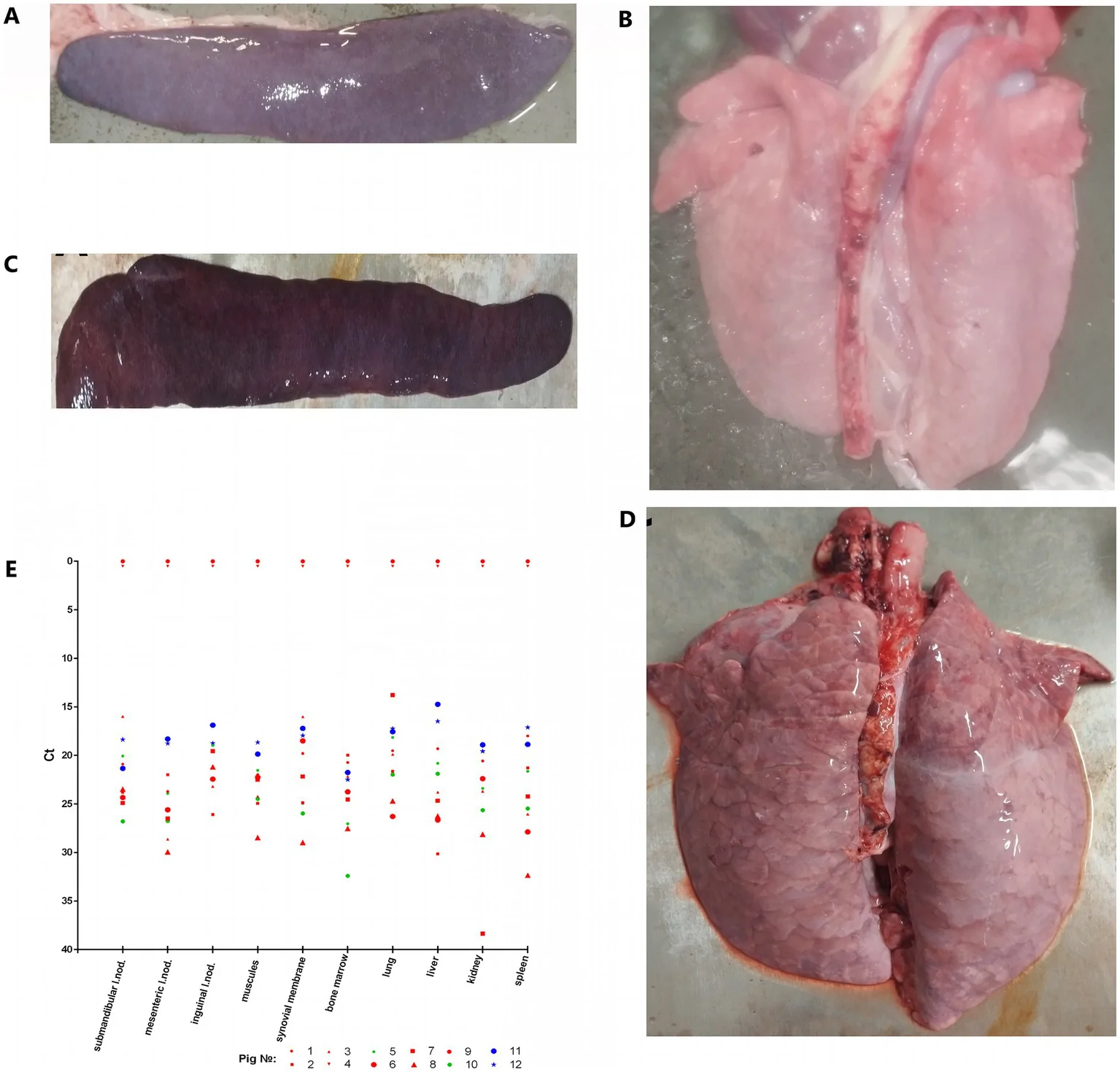
Pathological changes: **(A)** Spleen of recovered pig No. 4; **(B)** Lungs of recovered pig No. 9 after infection with the non-haemadsorbing isolate ASFV/Kirov/WB2023/1448 and subsequently challenged with the haemadsorbing isolate ASFV/Kirov/WB2023/1447. These animals underwent diagnostic slaughter on day 28 post-challenge infection; **(C)** Spleen of control pig No. 11; **(D)** Lungs of control pig No. 12 that died after infection with the isolate ASFV/Kirov/WB2023/1447. **(E)** Level of ASFV dissemination in various organs and tissues, as determined by real-time PCR (l.nod.—lymph node).

### Investigation of non-haemadsorbing virus circulation in the wild boar population

It has previously been demonstrated that infection with the non-haemadsorbing ASFV of genotype II (exemplified by isolate ASFV/Kirov/WB2023/1448) can result in the emergence of seropositive animals, both with and without detectable viraemia. To investigate the circulation of the attenuated virus in the wild, we present retrospective data from the analysis of samples collected from found-dead and hunted wild boars in Kirov and adjacent regions of the Russian Federation during the period 2021–2023, within a single population (which was also the source of isolates ASFV/Kirov/WB2023/1448 and ASFV/Kirov/WB2023/1447). A total of 122 samples of spleen, blood, serum, liver, and lymph nodes from the target wild boar population were tested in parallel by real-time PCR and IPT (for the detection of ASFV genome and specific antibodies, respectively) (Table 1).

**Table 1:** Results of a retrospective analysis of ASFV circulation among wild boars in the Kirov Region and bordering regions of Russia.

Region	2021			2022			2023		
	Total samples	Genome detected (real-time PCR)	Antibodies detected (IPT)	Total samples	Genome detected (real-time PCR)	Antibodies detected (IPT)	Total samples	Genome detected (real-time PCR)	Antibodies detected (IPT)
Kirov Region	–	–	–	–	–	–	31	7	6
Republic of Mari El	–	–	–	–	–	–	4	0	2
Republic of Tatarstan	8	8	0	1	0	0	–	–	–
Kostroma Region	60	0	0	3	3	3	–	–	–
Vologda Region	9	0	0	–	–	–	–	–	–
Arkhangelsk Region	–	–	–	6	6	1	–	–	–
Total	77	8	0	10	9	4	35	7	8

According to Table 1, the ASFV genome was detected in 19.7% (24/122) of the samples, whereas specific antibodies were identified in 9.8% (12/122).

All PCR-positive samples (24/122) were further tested by virus isolation. Haemadsorbing isolates were recovered in 23 out of 24 cases; only one sample (1/24) yielded the non-haemadsorbing ASFV/Kirov/WB2023/1448 isolate.

Antibodies were predominantly detected (10/12) in samples containing the ASFV genome. In only two exceptional cases (2/12), antibodies were found in PCR-negative samples collected in the Republic of Mari El in 2023. These two samples were additionally tested by virus isolation in PAM cell culture, with viral replication monitored by real-time PCR. No signs of virus replication were observed over five consecutive passages.

## Discussion

For the first time in the Russian Federation, during the current ASF epidemic (2007–present), a non-haemadsorbing variant of the ASFV has been identified. Prior to this study, only a few reports documented the detection of a non-haemadsorbing ASFV of genotype II in Eurasian countries: in Latvia (2017) and China (2020) (Gallardo et al., 2019; Sun et al., 2021). The characterized virus, ASFV/Kirov/WB2023/1448, is moderately virulent, causing up to 60% mortality and resulting in acute and subacute courses of ASF following both intramuscular and contact infection. In contrast, the Latvian strain Lv17/WB/Rie1 is a low-virulence, inducing chronic or asymptomatic disease, as well as prolonged (persistent) viraemia (Gallardo et al., 2019). The non-haemadsorbing strains from China are more similar in biological properties to ASFV/Kirov/WB2023/1448. However, moderate virulence comparable to that of the ASFV described in the current study was observed when pigs were infected with Chinese strains at a dose of 10^6^ TCID_50_. When animals were inoculated with a non-haemadsorbing virus at a dose of 10^6^ TCID_50_—as in the present experiment—no mortality was recorded, and the clinical signature was consistent with a low-virulence variant (Sun et al., 2021). Strain ASFV/Kirov/WB2023/1448 did not exhibit persistent viraemia (the maximum duration of virus detection in blood was 21 days), whereas the Latvian and Chinese non-haemadsorbing ASFV strains caused prolonged viraemia lasting up to 94 days. Furthermore, ASFV/Kirov/WB2023/1448 belongs to the genetic lineage «II/EU1/RU7», which is typical for Russia, while Lv17/WB/Rie1 belongs to «II/EU1/unclassified» and Chinese strains to «II/ASIA1/unclassified» (Figure 3D). These data indicate heterogeneity in the biological properties of non-haemadsorbing variants, even among ASFV isolates of genotype II derived from a common ancestor (Georgia 2007/1).

Despite the diversity of non-haemadsorbing variants, their detection remains limited to sporadic cases (Boinas et al., 2004; Leitão et al., 2001). In all cases, the *EP402R* gene, encoding the CD2v glycoprotein responsible for haemadsorption, is primarily subject to complete or partial deletion, thereby losing its primary function. Loss of haemadsorbing activity positively correlates with reduced duration of viraemia in pigs (Koltsov et al., 2023). The 3′-region of the *EP153R* gene, flanking *EP402R* at the 5′-end, is often co-deleted (Barasona et al., 2026). Deletion of *EP153R*, which encodes a C-type lectin-like protein, has a synergistic effect with deletion of *EP402R* in reducing ASFV virulence. However, a negative correlation between *EP153R* deletion and strain protectivity has been noted (Gladue et al., 2020; Lopez et al., 2021). In the present study, whole-genome analysis of ASFV/Kirov/WB2023/1448 revealed a large (~1,300 bp) deletion in the *EP153R* and *EP402R* genes. Similar mechanisms and patterns of natural attenuation of the ASFV in pigs underscore that the CD2v protein is one of the most important virulence factors.

Regarding the protective properties of CD2v-negative ASFV variants against highly virulent homologous genotype strains, data are varied and somewhat contradictory.

On one hand, non-haemadsorbing strains NH/P68 and OURT88/3 (genotype I) provided protection against infection with homologous ASFV (Boinas et al., 2004; Leitão et al., 2001; Sánchez-Cordón et al., 2017). Moreover, the double gene-deleted mutant ASFV-SY18-∆CD2v/UK, developed in China, protected immunized pigs from clinical signs of ASF when challenged with the parental highly virulent strain ASFV-SY18 (genotype II), but did not induce protection against viral persistence (after challenge infection, virulent ASFV continued to be detected in blood, oronasal and rectal swabs) (Teklue et al., 2020). A similar pattern was observed with strains Arm/07/CBM/c2 (co-deletion of *EP402R* (CD2v)/*A238L*), SY18ΔL60LΔCD2v (co-deletion of *EP402R* (CD2v)/*L60L*), and HuBΔCD2vΔA137R (co-deletion of *EP402R* (CD2v)/*A137R*) (Pérez-Núñez et al., 2022; Yang et al., 2024; Peng et al., 2025).

On the other hand, some studies demonstrate reduced protectivity of non-haemadsorbing ASFV. The initially attenuated strain Georgia-∆9GL, which induces protection against ASF, lost its protectivity following additional deletion of *EP402R* and *EP153R* genes (Lopez et al., 2021). Strain ASFV-Ke-ΔEP402RΔA238L provided only partial protection against challenge with the parental highly virulent strain ASFV-Ke (genotype IX) (Abkallo et al., 2022).

In the current study, animals that recovered from ASF following infection with ASFV/Kirov/WB2023/1448 were protected against challenge with the haemadsorbing virus ASFV/Kirov/WB2023/1447, which circulates in the same wild boar population as the non-haemadsorbing variant. No persistence of genotype II ASFV was observed in the recovered pigs, as evidenced by the absence of viraemia, virus shedding, and viral dissemination in organs and tissues. However, this study has several limitations due to the small animal sample size: 2 out of 10 pigs (no. 3 and 8) were euthanized because of secondary infections, and only 2 out of 10 pigs (no. 4 and 9) recovered and survived. Further research with a larger number of animals is needed to reach more reliable conclusions.

Previous studies on ASF seroprevalence in Russia reported the first detection of recovered wild boars in the Vladimir Region (Shotin et al., 2022). The data obtained from the assessment of the biological properties of the ASFV/Kirov/WB2023/1448 isolate demonstrated that some animals could survive ASFV infection, and did not rule out the possibility of circulation of variants with reduced virulence. This justified a retrospective analysis of sample data using real-time PCR, IPT, and virus isolation from wild boars collected in the same population where the non-haemadsorbing variant was detected. The identification of two seropositive individuals within the epidemic cluster (the Republic of Mari El borders Kirov Region and shares a common ecological system) in the absence of detectable ASFV may be considered as one piece of evidence supporting the hypothesis of circulation of a virus with attenuated properties, since survival of a part of the boar population after infection is possible. Notably, the wild boars presumed to have recovered from ASF were identified during the same period (the second half of 2023) when the non-haemadsorbing isolate ASFV/Kirov/WB2023/1448 was detected. Furthermore, in some boars (10/122) from Kostroma, Arkhangelsk, and Kirov Regions, both ASFV antibodies and the genome were detected, which may serve as a marker of subacute ASF—a pattern characteristic of the ASFV/Kirov/WB2023/1448 isolate. However, it should be noted that convincing evidence of such circulation would require a larger sample size in epidemiological studies within the wild boar population, as well as direct re-detection of the non-haemadsorbing variant in a subset of animals.

Thus, the results of this study may indicate the emergence of natural mechanisms leading to attenuation of ASFV in the European wild boar population over a relatively short period of pathogen circulation.

## Data Availability

The original contributions presented in the study are included in the article/supplementary material, further inquiries can be directed to the corresponding author.
